# Self‐Reported Versus Objectively Logged Social Media Use: Implications for Measurement in Eating Disorder Research

**DOI:** 10.1002/eat.70135

**Published:** 2026-05-31

**Authors:** Scott Griffiths, Emma Halliwell, Harish Kannan, Ben Stone, Wesley Grey, Noa Brittain, Eden Shaveet, Emily Harris

**Affiliations:** ^1^ Melbourne School of Psychological Sciences, University of Melbourne Melbourne Australia; ^2^ Unforgettable.Me Melbourne Australia; ^3^ Bowers College of Computing and Information Science, Cornell University New York USA

**Keywords:** eating disorders, measurement, mental health, social media, TikTok

## Abstract

**Objective:**

Self‐reported frequency measures of social media use (e.g., “How often do you use social media?”) are convenient, yet their criterion validity against objective behavioral data remains largely untested in eating disorder research. We compared self‐reports of TikTok use with objective data extracted from TikTok datafiles.

**Methods:**

Participants (*N* = 103) including individuals with diagnosed eating disorders (*n* = 42) completed a Likert‐type self‐report measure of TikTok use frequency over the past month. Objective data included the total number of TikTok videos delivered, total number of videos “liked” by participants, number of sessions, and number of “binge scroll” sessions (sessions lasting longer than 1 h). Associations were tested using Spearman correlations and negative binomial regressions.

**Results:**

Self‐reports were moderately to strongly correlated with objective indicators: videos delivered (*ρ* = 0.55), videos liked (*ρ* = 0.59), sessions (*ρ* = 0.65), and binge scroll sessions (*ρ* = 0.47). Importantly, objective use varied substantially within each self‐report response category. For example, among participants reporting they “sometimes” used TikTok over the past month, one was delivered 99 videos whereas another was delivered 19,526 videos, representing nearly a 200‐fold difference. Eating disorder diagnostic status and symptom severity did not moderate self‐report versus objective correspondence; however, statistical power to detect interaction effects was limited.

**Discussion:**

Self‐report frequency measures of social media are meaningfully correlated with objective indicators but compress substantial behavioral variability. Clinicians and researchers should interpret self‐reports cautiously, particularly when exposure magnitude is clinically relevant, and ought to consider objective data whenever possible.

## 1 Introduction

Social media has become a central feature of contemporary life and a major focus of psychological research, yet inferences about its mental health effects remain limited by how social media use is measured. A growing body of literature has examined associations between social media use and psychosocial outcomes, including eating disorder symptoms (Dondzilo et al. 2024; Griffiths et al. 2024), depression and loneliness (Twenge et al. 2018; Lin et al. 2016), and anxiety (Yan et al. 2017; Barry et al. 2017). However, findings remain inconsistent. Some studies report negative associations between social media use and mental health (Shakya and Christakis 2017; Huang 2022), others identify positive associations (Samad et al. 2019), and several report null effects (Best et al. 2014; Hall et al. 2021). Although platform evolution may partly contribute to this variability, methodological limitations, particularly how social media use is measured, are frequently cited as more plausible explanations (e.g., Keles et al. 2020). Establishing the validity of social media use measures is therefore essential for strengthening inference in this field.

### 1.1 Self‐Report Measures

Most studies examining social media use rely on self‐report measures (Ellis et al. 2019). Commonly, researchers assess frequency or duration of use, such as estimated time spent on social media. These frequency‐based measures are the focus of the present study. Strong conclusions regarding associations between social media use and mental health rely on the validity of frequency‐based self‐report measures (Scharkow 2016), and self‐reports have been criticized for their limited ability to capture actual behavior (Griffioen et al. 2020; Keles et al. 2020; Parry et al. 2021). Such inaccuracies may hinder replication, obscure true associations, and slow theoretical progress (Coyne et al. 2023). Consequently, researchers have begun to examine the correspondence between self‐reported and objectively logged behavioral social media data.

### 1.2 Types of Self‐Report Measures

Self‐report measures of social media use typically take three forms: open‐ended time estimates, categorical time estimates, and Likert‐type frequency ratings.

#### 1.2.1 Open‐Ended Time Estimates

Open‐ended time estimates require participants to estimate their time spent on social media over a specified period. A published example item is: “On average, how many hours a week do you think you spend viewing social media on your phone?” (Mahalingham et al. 2023). This format requires respondents to reconstruct multiple usage episodes across days, aggregate them, and convert fragmented interactions into a single temporal value. Evidence regarding validity is mixed. Coyne et al. (2023) compared estimated daily social media use with objectively logged time spent on social media and found a weak‐to‐moderate correlation (Pearson's *r* = 0.38), consistent with prior studies (Parry et al. 2021; Johannes et al. 2021; Ohme et al. 2021). In contrast, Mahalingham et al. (2023) reported a near‐zero correlation (*r* = 0.04) between estimated weekly iPhone social media use and screen time data.

Overall, open‐ended time estimates tend to show weak‐to‐moderate associations with logged duration and typically fall below thresholds considered sufficient to justify substituting subjective for objective indicators (Parry et al. 2021). Parry et al. (2021), in their meta‐analysis of the correlations between self‐reported and objectively logged digital media use, avoided providing a numerical threshold that would be considered “acceptable” for treating self‐reports and objective measures as equivalent. Nonetheless, in calculating a mean correlation of *r = 0*.38 (95% CI 0.33–0.42), Parry et al. (2021) concluded that “given the magnitude of the associations found in this meta‐analysis, the available evidence suggests that self‐reported measures should not be considered suitable substitutes for logs of social media use”. We agree with Parry et al. (2021) in this regard.

#### 1.2.2 Categorical Time Estimates

Categorical time estimates require participants to select from predefined time ranges rather than generate a precise numerical estimate. A published example item is: “How much time do you spend on your smartphone every day?” with response options including “Less than 1 hour”, “1–2 h”, “2–3 h”, “3–4 h”, and “More than 4 h” (Horwood et al. 2021). Although this format reduces the cognitive burden of calculating exact duration, it inherently introduces measurement imprecision because response categories are typically non‐equidistant. For example, the interval between “Less than 1 h” and “1–2 h” differs in magnitude from the interval between “3–4 h” and “More than 4 h,” yet these categories are often treated as if they are evenly spaced in quantitative analyses, compromising their interpretability (Furr 2021). Steele et al. (2022) reported small but significant correlations between categorical time estimates and objective metrics of total screentime on social media apps (*r* = 0.25), pick‐ups (*r* = 0.13), and notifications (*r* = 0.12).

#### 1.2.3 Likert‐Type Frequency Ratings

Likert‐type frequency ratings are arguably the most used method to assess how often individuals use social media platforms (Twenge et al. 2018; Shensa et al. 2017). A published example item is “How often have you used social media?” with response options including “never”, “rarely”, “sometimes”, “often”, and “constantly” (Griffiths, Castle, et al. 2018b). Despite their prevalence, relatively few studies have evaluated their correspondence with objectively logged behavioral data. Irmer and Schmiedek (2023) provide one of the only direct tests of this relationship: Participants rated the intensity of their daily use of Instagram, TikTok, and YouTube on a 5‐point Likert scale (1 = not at all to 5 = very much) and these were compared with objective app‐specific screen time data capturing logged duration of use. They reported moderate between‐person correlations between self‐reported ratings and logged screen time (Instagram: *r* = 0.36; TikTok: *r* = 0.58; YouTube: *r* = 0.49), as well as strong within‐person associations between daily self‐ratings and same‐day screen time (Instagram: *r* = 0.95; TikTok: *r* = 0.93; YouTube: *r* = 0.77). Compared to open‐ended time estimates (Verbeij et al. 2021; Boyle et al. 2022; Sewall et al. 2020), these findings suggest that Likert‐type frequency ratings may align more closely with logged behavior. However, further validation is required.

### 1.3 Objective Measures

Recent advancements in digital tracking technologies have enabled researchers to access participants' objective social media usage data (Ohme et al. 2021). Smartphone analytics such as Apple's Screen Time and Android's Digital Wellbeing provide app‐specific usage duration, total device time, pickups, and notifications (Coyne et al. 2023). The most commonly used objective indicator is smartphone screen time, which captures the logged duration of time spent on specific applications (Ohme et al. 2021). Although such metrics offer behavioral precision in estimating duration, they provide limited information about the nature of engagement (e.g., passive scrolling versus active interaction). Moreover, collecting mobile log data typically requires participants to manually locate, capture, and upload usage records, resulting in relatively low compliance rates (13%–35%) (Gower and Moreno 2018; Keusch et al. 2019). Low compliance may introduce systematic bias if individuals who share data differ from those who do not. Objective data collection also entails greater time and financial costs (Orben 2020). Thus, while objective measures improve behavioral precision, they introduce practical challenges. The key empirical question is how closely self‐reported estimates correspond to objectively logged use.

### 1.4 Social Media Measurement in the Context of Eating Disorders

Research linking the frequency of social media use to eating disorder symptomatology has grown rapidly and many studies in this area have relied on Likert‐type frequency ratings to assess frequency of social media use (e.g., Griffiths, Castle, et al. 2018b; Griffiths, Murray, et al. 2018a; Jarman et al. 2022; Holland and Tiggemann 2016; Wilksch et al. 2020; Yu et al. 2025). These measures are often acknowledged as a limitation because of the possibility that they might only weakly approximate actual behavior. However, to our knowledge, this assumption is yet to be tested with an eating disorders sample. If correspondence is weak or systematically distorted in individuals with eating disorders, then associations reported in the literature may reflect measurement artifact rather than true behavioral exposure. On the other hand, if self‐reported frequency ratings meaningfully correspond to objective behavioral data, then both researchers and clinicians can confidently proceed using self‐report questions.

#### 1.4.1 Eating Disorders and Reporting Accuracy

Despite extensive research on social media and eating disorder symptoms, no studies have examined whether eating disorder diagnosis or symptom severity influences the accuracy of self‐reported social media use, potentially confounding results. Retrospective estimates depend on attention, memory, and time perception (Grondin 2010), and may therefore be influenced by psychological characteristics (Sewall et al. 2020). Some evidence suggests that interoceptive deficits, characteristic of eating disorders, may affect time perception (Quadt et al. 2018; Brown et al. 2017). Interoception has been implicated in the development and maintenance of eating disorders (e.g., Jenkinson et al. 2018; Brown et al. 2017) and is associated with time estimation accuracy (Richter and Ibanez 2021). Meneguzzo et al. (2022) found diagnosis‐specific differences in time estimation: individuals with anorexia nervosa overestimated one‐minute intervals by approximately 9–10 s, individuals with binge eating disorder underestimated by approximately 1 s, and individuals with bulimia nervosa did not differ significantly from controls.

Research on other mental health conditions further indicates that reporting accuracy may vary by clinical status. Sewall et al. (2020) found that greater depressive symptoms were associated with greater inaccuracies in retrospective social media estimates, potentially due to cognitive and attentional impairments. Given overlapping cognitive features between depression and eating disorders (Puccio et al. 2016), similar distortions may plausibly occur among individuals with eating disorders. Understanding whether eating disorders influence reporting accuracy is therefore critical. If social media use is implicated in eating disorder symptomatology, measurement precision becomes central to both research validity and clinical interpretation.

### 1.5 Current Study: Aims and Hypotheses

Prior validation studies of social media use estimates have yielded inconsistent findings (Coyne et al. 2023; Mahalingham et al. 2023) and raised concerns regarding non‐equidistant response formats and measurement precision (Steele et al. 2022). Despite their widespread use in eating disorder research, Likert‐type frequency ratings remain comparatively underexamined relative to objective behavioral indicators (Irmer and Schmiedek 2023). Therefore, our study evaluates the criterion validity of a Likert‐type frequency rating of TikTok use against objectively logged behavioral indicators extracted directly from TikTok. Our focus is on between‐person correspondence: whether individuals who report using TikTok more frequently are, in fact, those who exhibit greater logged exposure and engagement. We focused on TikTok because it is one of the most widely used platforms among adolescents and young adults and has been the focus of recent research examining social media and eating disorder symptoms (e.g., Griffiths et al. 2024).

The primary aim of our study was to examine the strength of association between a Likert‐type frequency rating of TikTok use and objective digital trace indicators extracted directly from TikTok datafiles. Consistent with prior validation work, our confirmatory hypothesis was that positive associations would emerge between subjective and objective indicators of use, but we did not hypothesize how strong these associations would be. The secondary aim was to examine whether eating disorder characteristics influence the correspondence between subjective and objective use. Specifically, we tested whether (i) the presence of a current eating disorder diagnosis, and (ii) eating disorder symptom severity, moderate the association between self‐reported TikTok frequency and objective behavioral metrics. Based on theoretical work linking eating disorders to interoceptive differences and time estimation processes (Meneguzzo et al. 2022; Richter and Ibanez 2021), our exploratory hypothesis was that individuals with an eating disorder and those with higher symptom severity would show weaker correspondence between subjective and objective use.

## 2 Method

### 2.1 Participants

Participants (*N* = 103) were aged 16 to 38 years (*M*_age_ = 20.59, SD_age_ = 4.03) and were predominantly women (88%). Recruitment occurred between June 2022 and August 2023 via (i) Australian organizations supporting individuals with eating disorders (the Butterfly Foundation) and (ii) study advertisements distributed to psychology undergraduates at the University of Melbourne. The study was advertised as “a study of TikTok use” and the study aims and hypotheses were not disclosed. Participants received remuneration or course credit, with remuneration rates varying slightly depending on the quantity of TikTok data uploaded. Our remunerated participants were not aware of this slight variation—meaning, they were not incentivized to provide more data (i.e., watch more TikTok videos) than they otherwise would have. Inclusion criteria were: (i) age > 15 years, (ii) ownership of a smartphone, and (iii) English as the primary language used on TikTok. A minimum of 50 TikTok videos was an exclusion criterion designed to counteract potential fraudulent participants who may have created a TikTok account just so that they could participate in the study and receive remuneration. Two participants were excluded on this basis. Within the study sample, 42 participants reported a current eating disorder diagnosis (eating disorder group), and 49 reported no current or past eating disorder diagnosis or suspicion (healthy control group). The remainder (*n* = 12) reported a suspicion they had a current eating disorder but had not sought a formal diagnosis. Within the eating disorder group, 76.2% reported a formal diagnosis of anorexia nervosa.

### 2.2 Procedure

#### 2.2.1 Online Survey

Participants first completed an online survey assessing demographics, eating disorder diagnostic status, and eating disorder symptoms.

#### 2.2.2 Retrieval of Users' TikTok Data

Participants were then instructed to request their user data from TikTok. In Australia, user data access is governed by the Privacy Act 1988 (Australian Government 1988), specifically Australian Privacy Principle 12. TikTok's processing time ranged from 2 to 14 days (*M* = 4.5 days). Upon receipt, participants securely transmitted their TikTok data files to the research team.

#### 2.2.3 Data Processing and Metadata Scraping

TikTok user data files contained URLs for all videos delivered to participants' feeds, along with timestamps indicating when each video was delivered. Analyses were restricted to videos delivered within the month prior. A custom script was developed to scrape metadata from each video webpage linked in the dataset. Scraped metadata included whether the participant had “liked” the video. Pilot testing indicated perfect classification accuracy, with no observed false positives or false negatives. Objective indicators were then merged with participants' survey data (demographics, diagnostic history, eating disorder symptoms, and self‐reported TikTok use).

#### 2.2.4 Ethics and Conflict of Interest Statement

The study procedure was approved by the [masked for review] Human Research Ethics Committee (ID #23072). Informed consent was obtained from all participants.

### 2.3 Measures

#### 2.3.1 Diagnostic Status and Eating Disorder Symptoms

Lifetime eating disorder diagnosis was assessed with the item: “In your lifetime, have you ever been diagnosed with an eating disorder?” To ensure clarity, “diagnosed” was defined per Griffiths et al. (2015): “By ‘diagnosed’, we mean that a health professional (e.g., general practitioner or psychologist) has told you that you have an eating disorder.” Participants endorsing a lifetime diagnosis were subsequently asked whether they were currently diagnosed with an eating disorder. In our study, the eating disorders group comprises only those individuals who confirmed a current eating disorder diagnosis. Eating disorder symptom severity was assessed using the Eating Pathology Symptom Inventory (EPSI; Forbush et al. 2013), which demonstrates strong psychometric properties. Participants reported symptoms over the past month, aligning with the one‐month TikTok activity window. Higher scores reflected greater symptom severity. Analyses used the global EPSI score, hereafter referred to as eating disorder symptoms.

#### 2.3.2 Self‐Reported TikTok Use

Self‐reported TikTok use was assessed with a single item: “In the past month, how often have you used TikTok?” Responses were recorded on a five‐point Likert‐type scale ranging from 1 (never) to 5 (constantly). We selected a Likert‐type frequency item rather than an open‐ended time estimate (e.g., hours per day) or categorical time‐range measure because Likert‐type scales are commonly used and avoid several limitations of alternative formats. Open‐ended time estimate measures require participants to generate precise temporal calculations, which have shown the weakest and most inconsistent correspondence with objective data. Categorical time estimates, although easier to answer, involve non‐equidistant response intervals that complicate quantitative interpretation. In contrast, Likert‐type scales assess perceived intensity or frequency without requiring explicit time estimation, making them both theoretically relevant and psychometrically pragmatic.

#### 2.3.3 Objective TikTok Use

Using the TikTok user data files and scraped metadata, four objective indicators of use were calculated for the one‐month observation window: (i) the total number of videos delivered to each participant's feed, (ii) the total number of videos “liked” by each participant, (iii) the total number of sessions of use, and (iv) the number of “binge scrolling” sessions. A “session” was operationalized as a continuous period of TikTok activity. Consecutive video deliveries were grouped into the same session unless there was a gap of five or more minutes between them; such a gap marked the end of one session and the start of another. Although TikTok videos longer than 5 min are rare, we conducted sensitivity analyses using alternative inactivity thresholds of 10 and 15 min. These yielded substantively similar results; therefore, all reported analyses use the five‐minute threshold. **“**Binge scrolling”, referring to prolonged, continuous scrolling through content (Park and Jung 2024), was operationalized as sessions lasting longer than one hour. Currently, there is no standardized duration threshold for binge scroll behavior in digital media research and operationalizations vary widely across studies. We selected a one‐hour threshold as a sensible sounding boundary to distinguish an extended (i) session of use from a “regular” session of use.

#### 2.3.4 Age and Gender

We asked participants to indicate their age using a drop‐down list to avoid typos. We assessed gender by asking participants to select the option that best describes their gender, with options, “Cisgender woman (I am a woman and I was assigned female at birth)”, “Cisgender man (I am a man and I was assigned male at birth)”, “Something else (if you select this option, a list of different genders will appear)”, and “I'd prefer not to say.”

### 2.4 Data Analyses

Prior to analysis, data were screened and cleaned. Participants with missing values on primary variables (self‐reported TikTok use, objective TikTok use, eating disorder diagnosis, or eating disorder symptoms) were excluded. Twenty‐one participants who suspected but had not received a formal eating disorder diagnosis were excluded from group‐based analyses but retained for symptom‐based analyses. Objective TikTok use indicators (videos delivered, videos liked, sessions, and binge scroll sessions) were positively skewed and highly overdispersed (variance substantially exceeding the mean). Given the count‐based and overdispersed nature of these outcomes, Spearman's correlations and negative binomial regression models were used instead of Pearson correlations and linear regression models. All analyses were conducted using R (Version 2024.12.1).

#### 2.4.1 Primary, Confirmatory Hypothesis

To examine the association between self‐reported TikTok use and objective indicators of TikTok activity, we conducted Spearman's rank‐order correlations to assess the associations between the single‐item Likert‐type self‐report measure and each objective count variable (videos delivered, videos liked, sessions, and binge scroll sessions).

#### 2.4.2 Secondary, Exploratory Hypothesis

To examine whether eating disorder characteristics moderated the association between self‐reported and objective TikTok use, interaction effects were tested using negative binomial regression models. In each model, self‐reported TikTok use was entered as the predictor variable and one objective indicator was entered as the outcome variable.

We used two different operationalizations of eating disorder status as the moderator: (i) the presence of a self‐reported eating disorder diagnosis (i.e., eating disorder versus healthy controls); and (ii) eating disorder symptom severity (as measured by the EPSI). In the former operationalization, eating disorder diagnosis (0 = healthy control; 1 = current eating disorder diagnosis) was entered as a categorical moderator of the relationship between self‐reported TikTok use and each objective outcome. In the symptom‐based analyses, eating disorder symptom severity (global EPSI score) was entered as a continuous moderator. For these models, self‐reported TikTok use and EPSI scores were mean‐centered prior to analysis to reduce multicollinearity and facilitate interpretation of interaction effects. The interaction term was then specified as the product of the centered predictor and moderator variables. Each model included the main effects of self‐reported TikTok use and the moderator, as well as their interaction term.

All moderation models were adjusted for age and gender to account for potential demographic covariation with TikTok use. Gender was coded as a binary variable (man, woman). One participant who identified as non‐binary was excluded from the analysis. While conducting our negative binomial regressions, we monitored for multicollinearity among predictors by assessing variance inflation factors (VIFs) and tolerance statistics and observed no evidence of problematic multicollinearity (i.e., VIFs > 5, tolerances < 0.20). We also ran a sensitivity analysis including only individuals diagnosed with anorexia nervosa; results from these models were substantively identical and are not further discussed.

#### 2.4.3 Power Analyses

Power for Spearman's correlations was evaluated post hoc via Monte Carlo simulation using a Gaussian copula approach (bivariate normal data, converted to ranks), two‐tailed *α* = 0.05, and *N* = 103. Results showed that our power was approximately 0.98 to detect *ρ* = 0.40, 0.84 for *ρ* = 0.30, 0.48 for *ρ* = 0.20, and 0.26 for *ρ* = 0.10, indicating adequate power to detect moderate‐to‐large sized associations, but low power to detect small‐to‐moderate interaction effects. Similarly, for the negative binomial regressions, simulations suggested we were adequately powered to detect moderate‐to‐large main effects (Incidence rate ratios [IRRs] of 1.30 and greater).

## 3 Results

### 3.1 Descriptive Statistics

**FIGURE 1:**
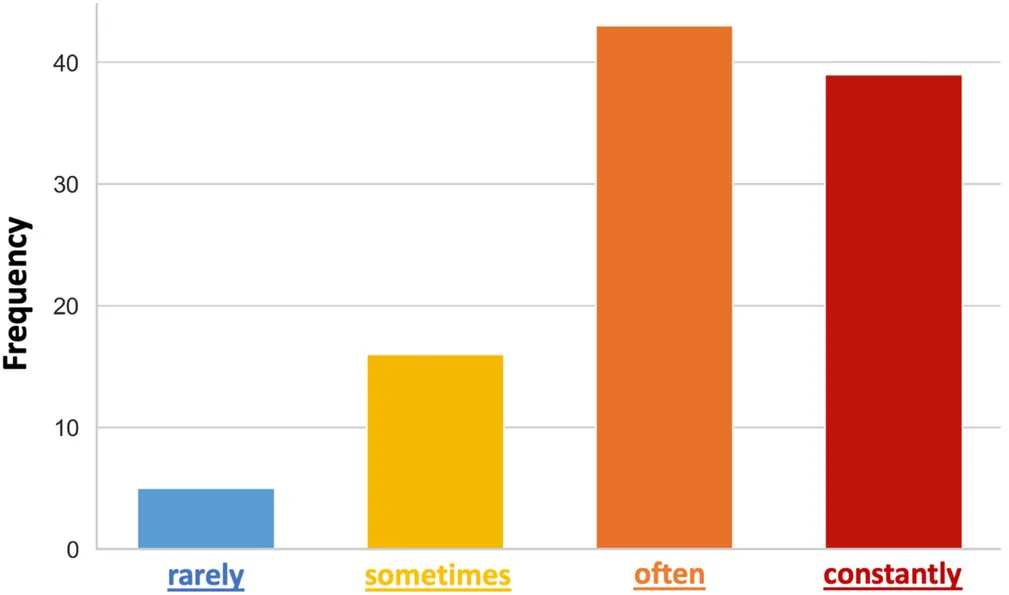
Frequency distribution of self‐reported frequency of TikTok use. The color of each bar is matched to Figure 2.

**FIGURE 2:**
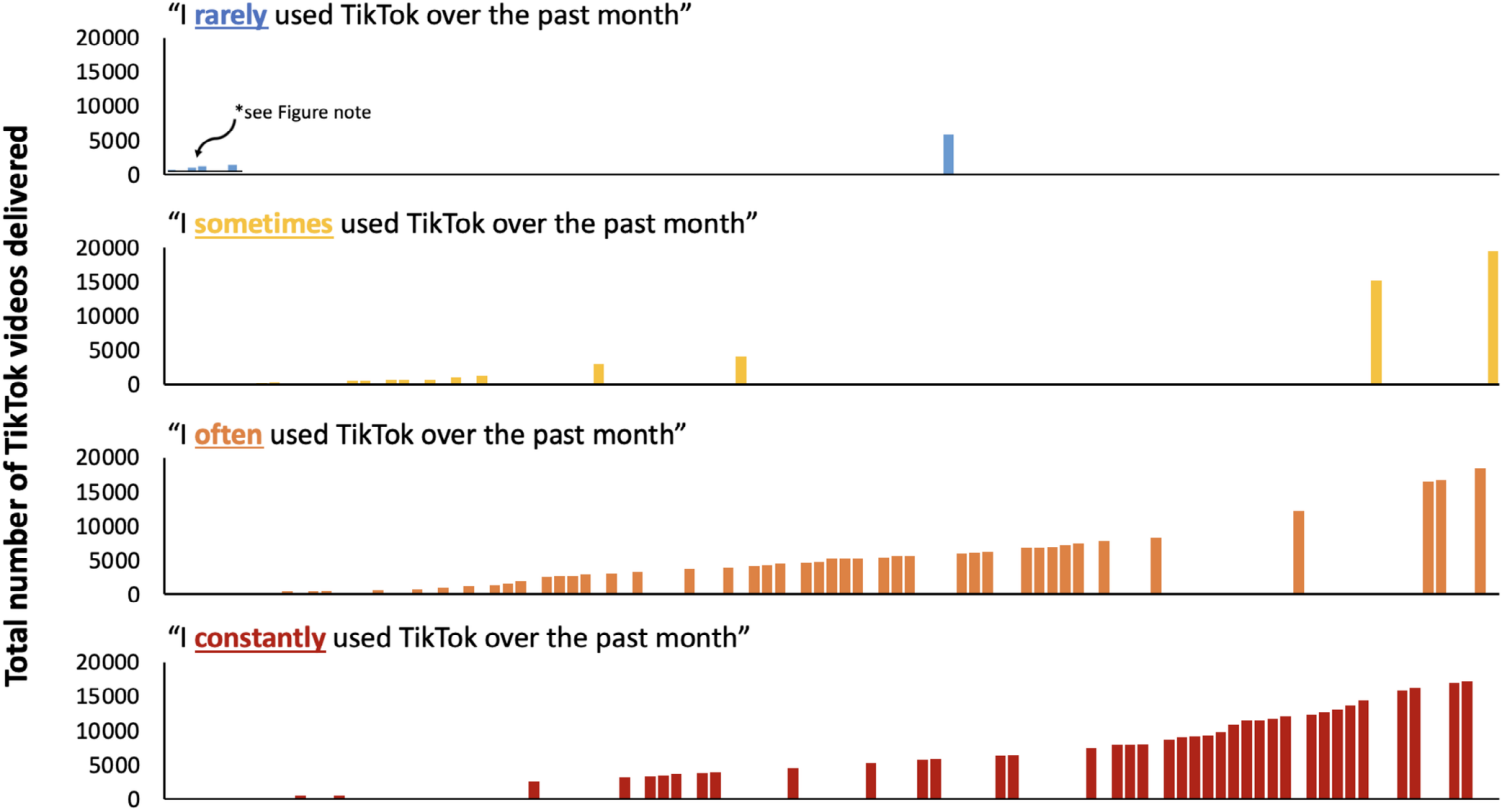
Distribution of objective TikTok video deliveries by self‐reported frequency of use. Each column represents one participant's total number of TikTok videos delivered over the past 30 days (*y*‐axis), derived from objective digital trace data. Panels are grouped by participants' self‐reported frequency of TikTok use (“rarely,” “sometimes,” “often,” and “constantly”). Participants are ordered within each panel by increasing number of videos delivered to illustrate within‐category variability. *Data columns for four participants who received 62, 132, 160, and 206 videos, respectively, have been enlarged for visibility.

Table 1 provides summary statistics for self‐reported frequency of TikTok use, objectively determined TikTok use, and eating disorder symptoms. As shown in Figure 1, participants generally reported high levels of TikTok use with responses tightly clustered around 4 (“often”) and many participants reporting the maximum of 5 (“constantly”). By contrast, participants' objective indicators of TikTok were much more variable. Figure 2 shows the total number of videos delivered for participants who reported that they rarely, sometimes, often, and constantly used TikTok over the past month. Within each self‐report response option, the variability in participants' objectively determined number of TikTok videos delivered was conspicuously high. For example, among participants who self‐reported that they “sometimes” used TikTok over the past month (see the yellow columns), our objective data showed that one participant was delivered 99 videos whereas another was delivered 19,526 videos. This pattern was consistent across our other dependent variables: number of “liked” videos, number of sessions, and number of binge scroll sessions.

**TABLE 1:** Descriptive statistics and Spearman correlation coefficients for self‐reported TikTok use, objective TikTok use, and eating disorder symptoms.

Variable	*M*	*Mdn*	SD	Range	Spearman's correlation coefficient (*ρ*)					
					1	2	3	4	5	6
1. Self‐reported frequency of TikTok use	4.13	4	0.85	2–5	—	0.55*	0.59*	0.65*	0.40*	0.18
2. Objective number of TikTok videos delivered	6069.73	5223	5066.87	62–19,526		—	0.85*	0.83*	0.77*	−0.01
3. Objective number of liked TikTok videos	1623.68	1256	1423.87	6–6113			—	0.84*	0.66*	0.03
4. Objective number of TikTok sessions	171.40	169	121.56	3–491				—	0.56*	0.03
5. Objective number of binge scroll sessions	7.75	3.00	10.59	0–46					—	−0.06
6. Eating disorder symptoms	2.40	2.40	0.60	1.13–4.02						—

*Note:* *p < 0.001. Self‐reported TikTok use was measured using a single‐item Likert scale ranging from 1 (never) to 5 (constantly). Objective TikTok use variables were derived from participant‐requested TikTok data files and subsequent metadata scraping. The time window for all measures was 1 month.

### 3.2 Primary, Confirmatory Hypothesis: Spearman Correlations

The Spearman correlations shown in Table 1 indicate significant, moderate‐sized positive monotonic correlations between self‐reported TikTok use and all four objective indicators of platform activity. Specifically, self‐reported frequency was significantly correlated with the number of videos delivered, number of videos liked, number of sessions, and number of binge scroll sessions (all *p*s < 0.001), suggesting that participants who reported more frequent use generally exhibited higher objectively determined use. All four objective measures were strongly intercorrelated (*p*s < 0.001).

In contrast, eating disorder symptoms were not significantly associated with either self‐reported TikTok use or any objective use metrics, except for binge scroll sessions. In three of our four regression models, frequency of binge scrolling was associated with fewer eating disorder symptoms and a lower likelihood of having an eating disorder diagnosis.

### 3.3 Secondary, Exploratory Hypothesis: Negative Binomial Regressions

Table 2 provides summary statistics for the negative binomial regression models examining whether eating disorder diagnostic status (diagnosed versus healthy controls) moderates the strength of the associations between self‐reported and objectively determined TikTok use. Table 3 provides summary statistics for the same regression with two co‐variates added: age (in years) and gender (man/woman). Table 4 provides summary statistics for the negative binomial regression models examining whether eating disorder symptom severity (measured by the EPSI) moderates the strength of the associations between self‐reported and objectively determined TikTok use. Table 5 provides summary statistics for the same regression with two co‐variates added: age (in years) and gender (man/woman).

**TABLE 2:** Eating disorder diagnosis model summaries: Results from the negative binomial regression models using diagnosis (healthy controls versus eating disorders), subjective TikTok use, and the interaction of diagnosis and subjective use to predict four indicators of objective TikTok use.

Objective TikTok use	Predictor	b (95% CI)	SE_b_	IRR (95% CI)	Wald *χ* ^2^, df = 1	*p*
Number of videos delivered	Subjective use	1.07 (0.60, 1.53)	0.24	2.91 (1.83, 4.61)	20.45	< 0.001
	Diagnosis^a^	0.26 (−0.18, 0.69)	0.22	1.29 (0.84, 1.99)	1.33	0.249
	Interaction	−0.38 (−0.99, 0.23)	0.31	0.68 (0.37, 1.26)	1.51	0.219
Number of videos liked	Subjective use	0.99 (0.59, 1.38)	0.21	2.69 (1.81, 3.99)	23.79	< 0.001
	Diagnosis^a^	0.15 (−0.29, 0.58)	0.22	1.16 (0.75, 1.78)	0.43	0.510
	Interaction	−0.16 (−0.73, 0.41)	0.29	0.85 (0.48, 1.51)	0.30	0.583
Number of sessions	Subjective use	1.09 (0.68, 1.51)	0.21	2.99 (1.97, 4.53)	26.45	< 0.001
	Diagnosis^a^	0.37 (−0.07, 0.80)	0.22	1.44 (0.93, 2.22)	2.71	0.100
	Interaction	−0.43 (−0.99, 0.14)	0.29	0.65 (0.37, 1.15)	2.19	0.139
Number of binge scrolls	Subjective use	0.93 (0.38, 1.49)	0.28	2.54 (1.46, 4.42)	10.86	< 0.001
	Diagnosis^a^	0.35 (−0.12, 0.83)	0.24	1.42 (0.89, 2.28)	2.11	0.146
	Interaction	−0.27 (−0.96, 0.42)	0.35	0.76 (0.38, 1.53)	0.58	0.446

*Note:* Healthy control is the reference level. b = unstandardized regression coefficient.

Abbreviations: CI, confidence interval; df, degrees of freedom; IRR, Incidence Rate Ratio; SE, standard error.

^a^ Diagnosis is a categorical variable with two levels: 0 = healthy control, 1 = eating disorder.

**TABLE 3:** Eating disorder symptom model summaries: Results from the negative binomial regression models using eating disorder symptoms, subjective TikTok use, and the interaction of symptoms and subjective use to predict four indicators of objective TikTok use.

Objective TikTok use	Predictor	b (95% CI)	SE_b_	IRR (95% CI)	Wald *χ* ^2^, df = 1	*p*
Number of videos delivered	Subjective use	0.60 (0.36, 0.83)	0.13	1.82 (1.43, 2.30)	24.51	< 0.001
	Symptoms	−0.31 (−0.69, 0.07)	0.19	0.74 (0.50, 1.07)	2.54	0.111
	Interaction	0.23 (−0.27, 0.73)	0.26	1.26 (0.76, 2.08)	1.26	0.370
Number of videos liked	Subjective use	0.82 (0.56, 1.08)	0.14	2.27 (1.75, 2.95)	37.51	< 0.001
	Symptoms	−0.40 (−0.79, 0.00)	0.20	0.67 (0.45, 1.00)	3.85	0.050^†^
	Interaction	0.46 (−0.11, 1.03)	0.29	1.59 (0.90, 2.81)	2.49	0.114
Number of sessions	Subjective use	0.66 (0.42, 0.90)	0.12	1.93 (1.52, 2.46)	28.68	< 0.001
	Symptoms	−0.23 (−0.62, 0.17)	0.20	1.23 (0.54, 1.18)	1.28	0.259
	Interaction	0.25 (−0.27, 0.76)	0.27	1.28 (0.76, 2.15)	0.85	0.355
Number of binge scrolls	Subjective use	0.77 (0.48, 1.05)	0.14	2.15 (1.62, 2.85)	28.07	< 0.001
	Symptoms	−0.63 (−1.04, −0.22)	0.21	0.53 (0.35, 0.80)	9.18	0.002
	Interaction	0.37 (−0.18, 0.92)	0.28	1.44 (0.83, 2.50)	1.71	0.192

*Note:* b = unstandardized regression coefficient, ^†^ *p* = 0.0499 at 4 decimal places, but we have interpreted the value as non‐significant given it is 0.050 at 3 decimal 1places.

Abbreviations: CI, confidence interval; df, degrees of freedom; IRR, Incidence Rate Ratio; SE, standard error.

**TABLE 4:** Eating disorder diagnosis model summaries with covariates: Results from the negative binomial regression models using diagnosis (healthy controls versus eating disorders), subjective TikTok use, and the interaction of diagnosis and subjective use to predict four indicators of objective TikTok use, including age and gender as co‐variates.

Objective TikTok use	Predictor	b (95% CI)	SE_b_	IRR (95% CI)	Wald *χ* ^2^, df = 1	*p*
Number of videos delivered	Subjective use	1.11 (0.66, 1.56)	0.23	3.04 (1.94, 4.76)	23.64	< 0.001
	Diagnosis^a^	−0.23 (−0.69, 0.23)	0.23	0.80 (0.50, 1.26)	0.95	0.329
	Interaction	−0.21 (−0.85, 0.43)	0.33	0.80 (0.43, 1.54)	0.41	0.522
	Age (years)	0.01 (−0.04, 0.05)	0.02	1.01 (0.96, 1.05)	0.08	0.779
	Gender^b^	0.59 (−0.09, 1.26)	0.35	1.80 (0.91, 3.53)	2.86	0.091
Number of videos liked	Subjective use	0.97 (0.56, 1.39)	0.21	2.64 (1.74, 4.00)	21.03	< 0.001
	Diagnosis^a^	−0.01 (−0.46, 0.45)	0.23	0.99 (0.64, 1.59)	< 0.01	0.968
	Interaction	−0.01 (−0.64, 0.63)	0.32	0.99 (0.53, 1.88)	< 0.01	0.985
	Age (years)	−0.05 (−0.09, 0.00)	0.02	0.96 (0.91, 1.00)	3.65	0.056
	Gender^b^	0.34 (−0.32, 1.01)	0.34	1.42 (0.73, 2.73)	1.03	0.311
Number of sessions	Subjective use	1.07 (0.64, 1.50)	0.22	2.92 (1.90, 4.49)	23.92	< 0.001
	Diagnosis^a^	0.27 (−0.20, 0.74)	0.24	1.31 (0.82, 2.09)	1.29	0.255
	Interaction	−0.30 (−0.92, 0.32)	0.32	0.74 (0.40, 1.38)	0.91	0.341
	Age (years)	−0.02 (−0.06, 0.03)	0.02	0.98 (0.94, 1.03)	0.63	0.429
	Gender^b^	0.29 (−0.41, 0.98)	0.35	1.33 (0.62, 2.66)	0.65	0.422
Number of binge scrolls	Subjective use	1.42 (0.76, 2.07)	0.33	4.13 (2.15, 7.93)	18.06	< 0.001
	Diagnosis^a^	0.66 (0.11, 1.22)	0.28	1.94 (1.11, 3.39)	5.42	0.020
	Interaction	−0.28 (−1.04, 0.48)	0.39	0.76 (0.35, 1.62)	0.52	0.469
	Age (years)	0.06 (0.00, 0.12)	0.03	1.06 (1.00, 1.12)	4.40	0.036
	Gender^b^	−1.22 (−2.00, −0.44)	0.40	0.30 (0.14, 0.65)	9.36	0.002

*Note:* Healthy control is the reference level. Woman is the reference level. b = unstandardized regression coefficient.

Abbreviations: CI, confidence interval; df, degrees of freedom; IRR, Incidence Rate Ratio; SE, standard error.

^a^ Diagnosis is a categorical variable with two levels: 0 = healthy control, 1 = eating disorder.

^b^ Gender is a categorical variable with two levels: 0 = woman, 1 = man.

**TABLE 5:** Eating disorder symptom model summaries with covariates: Results from the negative binomial regression models using eating disorder symptoms, subjective TikTok use, and the interaction of symptoms and subjective use to predict four different indicators of objective TikTok use, including age and gender as co‐variates.

Objective TikTok use	Predictor	b (95% CI)	SE_b_	IRR (95% CI)	Wald *χ* ^2^, df = 1	*p*
Number of videos delivered	Subjective use	0.62 (0.37, 0.87)	0.13	1.85 (1.44, 2.38)	23.30	< 0.001
	Symptoms	−0.30 (−0.68, 0.08)	0.19	0.74 (0.51, 1.09)	2.34	0.126
	Interaction	0.19 (−0.33, 0.71)	0.27	1.21 (0.72, 2.04)	0.52	0.471
	Age (years)	−0.02 (−0.06, 0.02)	0.02	0.98 (0.94, 1.02)	1.05	0.306
	Gender^a^	0.13 (−0.47, 0.72)	0.31	1.13 (0.62, 2.06)	0.17	0.681
Number of videos liked	Subjective use	0.83 (0.55, 1.11)	0.14	2.30 (1.74, 3.05)	34.16	< 0.001
	Symptoms	−0.35 (−0.75, 0.04)	0.20	0.70 (0.47, 1.04)	3.10	0.078
	Interaction	0.37 (−0.23, 0.97)	0.30	1.45 (0.80, 2.63)	1.49	0.211
	Age (years)	−0.04 (−0.08, 0.00)	0.02	0.96 (0.92, 1.00)	3.15	0.076
	Gender^a^	0.08 (−0.55, 0.70)	0.32	1.08 (0.50, 1.73)	0.06	0.810
Number of sessions	Subjective use	0.67 (0.41, 0.94)	0.13	1.96 (1.51, 2.55)	25.69	< 0.001
	Symptoms	−0.20 (−0.60, 0.19)	0.20	0.82 (0.55, 1.21)	0.99	0.320
	Interaction	0.19 (−0.37, 0.74)	0.28	1.21 (0.69, 2.10)	0.44	0.506
	Age (years)	−0.04 (−0.08, 0.00)	0.02	0.96 (0.92, 1.00)	3.15	0.076
	Gender^a^	0.08 (−0.55, 0.70)	0.32	1.08 (0.50, 1.73)	0.06	0.810
Number of binge scrolls	Subjective use	0.87 (0.56, 1.18)	0.16	2.39 (1.76, 3.26)	30.78	< 0.001
	Symptoms	−0.64 (−1.05, −0.23)	0.21	0.53 (0.35, 0.79)	9.43	0.002
	Interaction	0.32 (−0.24, 0.89)	0.29	1.38 (0.79, 2.43)	1.27	0.261
	Age (years)	0.00 (−0.04, 0.05)	0.02	1.00 (0.96, 1.05)	< 0.01	0.953
	Gender^a^	−0.61 (−1.27, 0.05)	0.34	0.54 (0.28, 1.05)	3.32	0.068

*Note:* Woman is the reference level. b = unstandardized regression coefficient.

Abbreviations: CI, confidence interval, df, degrees of freedom; RR, Incidence Rate Ratio; SE, standard error.

^a^ Gender is a categorical variable with two levels: 0 = woman, 1 = man.

Across all negative binomial models, there was no evidence that eating disorder characteristics moderated the association between self‐reported and objective TikTok use. In the diagnosis‐based analyses, the interaction between subjective TikTok use and eating disorder diagnosis was non‐significant for all four objective indicators including videos delivered, videos liked, sessions, and binge scrolling sessions. Similarly, in the symptom‐based analyses, the interaction between symptom severity and mean‐centered self‐reported use was consistently non‐significant, suggesting that eating disorder symptom severity did not alter the correspondence between subjective and objective TikTok use.

The co‐variates age and gender showed limited and outcome‐specific associations. Age was unrelated to videos delivered, liked, or total sessions, but was positively associated with binge scroll sessions in one regression model, suggesting slightly greater extended use among older participants. In one regression model, females engaged in more binge scrolling than males. Given these results are uncorrected for multiple comparisons, our holistic interpretation is that neither age nor gender is meaningfully related to our primary research question concerning the correspondence between self‐reported and objectively determined measures of TikTok use.

## 4 Discussion

The primary aim of our study was to examine the strength of the association between self‐reported and objectively measured TikTok use. Our secondary aim was to examine whether this association was moderated by eating disorder diagnosis or symptom severity. We found that self‐reported TikTok use was moderately to strongly associated with all objective indicators, including videos delivered, videos liked, sessions, and binge scroll sessions. However, these correlation coefficients concealed substantial dispersion in the objective data, which carries implications both for researchers wishing to use self‐report frequency measures and for clinicians enquiring about clients' social media use. Regarding our secondary aim, we found no evidence that eating disorder diagnosis or symptom severity moderated the strength of the association between self‐reported TikTok use and objectively measured TikTok use, suggesting that individuals with eating disorders are as accurate or inaccurate as healthy control participants when recalling their social media use. Limited statistical power is an important caveat to this latter finding.

### 4.1 Associations Between Self‐Reported and Objective TikTok Use

Our single‐item 5‐point Likert‐type frequency rating of TikTok use was moderately to strongly associated with objectively extracted TikTok metadata, including the number of videos delivered, number of videos liked, number of sessions, and number of binge scroll sessions, with Spearman correlations ranging from *ρ* = 0.47 to 0.65. This pattern aligns with Irmer and Schmiedek (2023), who reported correlations between Likert‐type social media frequency ratings and device‐logged smartphone screen time in the range of *r* = 0.50–0.70. By contrast, our correlations are stronger than studies using open‐ended time estimates, which have typically reported weak‐to‐moderate correlations with device‐recorded screen time and app usage duration time, often in the range of *r* = 0.20–0.40 (Coyne et al. 2023; Parry et al. 2021; Johannes et al. 2021; Ohme et al. 2020; Mahalingham et al. 2023). Likert‐type frequency ratings may outperform open‐ended time estimates because they require respondents to make broad, ordinal judgments about how often they use a platform (e.g., “rarely” to “constantly”) rather than to retrospectively calculate the number of minutes or hours spent using it. Estimating duration requires reconstructing multiple usage episodes, summing them across days, and converting fragmented interactions into a single numeric value. This process places substantial cognitive demands on memory and is highly vulnerable to rounding, telescoping (misplacing events in time), and heuristic shortcuts. In contrast, frequency‐based ratings ask participants to position their behavior relative to their own typical use, which relies more on global impressions than on precise temporal reconstruction. By reducing the need for exact duration recall, Likert‐type frequency ratings may minimize systematic estimation error and produce stronger correspondence with objective behavioral logs.

Despite moderate‐to‐strong correlations, substantial dispersion was observed in objective TikTok use. Self‐reported frequency ratings were concentrated toward the upper end of the 5‐point scale, whereas the objective indicators showed marked overdispersion. Participants who selected the same Likert category often differed dramatically in their recorded behavior. For example, among those who reported “sometimes” using TikTok over the past month, one participant was delivered 99 videos whereas another was delivered 19,526 videos. This nearly 200‐fold difference illustrates that although the scale captures relative ordering, it collapses large quantitative differences into the same ordinal category. This compression of variability may be problematic in research contexts where the magnitude of exposure matters. If outcomes are hypothesized to follow dose–response relationships, threshold effects, or non‐linear associations, a coarse frequency scale may obscure clinically or theoretically meaningful differences. Two individuals both classified as using TikTok “often” may differ by thousands of delivered or “liked” videos, and/or by hundreds of sessions, potentially leading to attenuation or exaggeration of effect sizes or failure to detect high‐risk subgroups. In policy or clinical research, where identifying extreme levels of use is important, such compression could mask meaningful heterogeneity, lead to inaccurate effect sizes, and produce inconsistency in the literature.

However, this limitation is less consequential in research designs focused on relative standing or within‐person change. In repeated‐measures or intervention studies where participants are compared against their own baseline, a Likert‐type frequency rating may adequately capture directional shifts over time (e.g., from “often” to “sometimes”), even if it does not reflect exact exposure magnitude. Though, empirical work is needed to confirm whether participants' definitions of response categories such as “sometimes” remains roughly consistent over time. Similarly, in large‐scale epidemiological studies where social media use functions as a contextual covariate rather than a primary exposure, ordinal frequency ratings may provide a pragmatic and sufficiently informative proxy.

### 4.2 Eating Disorders and Self‐Reporting Accuracy

Eating disorder diagnosis and symptom severity did not moderate the association between self‐reported and objective TikTok use, meaning that individuals with eating disorders were neither more nor less accurate in reporting their general TikTok frequency relative to healthy controls. This pattern was consistent across categorical and continuous operationalizations of eating disorder status. Prior research has suggested that psychological factors may influence time perception (Meneguzzo et al. 2022) and retrospective reporting accuracy (Sewall et al. 2020). However, the big caveat to these findings is statistical power, which was modest in our study and limited our ability to detect interaction effects. Interaction effects are typically smaller than main effects and require larger samples to detect reliably (Blake and Gangestad 2020; Whisman and McClelland 2005). We can, however, suggest that eating disorder symptoms and diagnostic status do not appear to have a strong/large moderating effect on the concordance between self‐reported and objectively determined TikTok use.

Statistical power notwithstanding, one speculative explanation for the absence of moderation effects concerns schema‐consistent memory processes. Individuals with eating disorders often demonstrate attentional and memory biases toward disorder‐relevant information (Pona et al. 2019; Lee and Shafran 2004). Given evidence that individuals with eating disorders may be disproportionately exposed to eating disorder–related content online (Griffiths et al. 2024), heightened salience of such material could preserve recall of general usage frequency. Future research might examine whether the association between self‐report and objectively logged social media use is stronger among those with eating disorders to the extent that disorder‐relevant content (e.g., dieting videos) makes up a larger amount and/or volume of the content delivered by their algorithm.

### 4.3 Clinical Implications

Clinical assessment focuses on the individual rather than population‐level trends, and at this level the extreme dispersion in objective use concealed within self‐report response categories is consequential. As shown in Figure 1, some participants endorsing the same Likert response option differed in their actual use by many thousands of videos. Although not visualized, this was also true for our other objective indicators: videos liked, sessions, and binge scrolling sessions. Independent of debates about whether more frequent social media use is empirically predictive of eating disorder symptoms and other psychological phenomena, we believe most clinicians would reasonably wish to know whether a client answer of “sometimes” reflects relatively modest or near‐continuous exposure. When clinicians have reason to believe that social media could be clinically relevant to their client with an eating disorder, we recommend follow‐up questions beyond “How often do you use social media?” that probe, for example, exposure volume and number of sessions. For example: “Yesterday, how many times did you open TikTok?” “Was that a typical day for you?” “Do you usually open TikTok more or less than that?” Per the data in Table 1, some participants in our study were opening TikTok only once or twice a week, while others opened TikTok multiple times per day. Unfortunately, TikTok and other major social media players do not make detailed usage metadata of the kind we have analyzed here available to end‐users. Until such data are routinely available, subjective frequency ratings should be interpreted cautiously, with recognition that identical responses may mask profoundly different levels of behavioral exposure.

### 4.4 Study Limitations

Several limitations warrant consideration. First, the self‐report item referenced TikTok use over the past month. Longer recall windows may attenuate correspondence due to memory decay, aggregation biases, and reduced sensitivity to day‐to‐day variation (Timotijevic et al. 2009; Irmer and Schmiedek 2023). Second, the restricted range of the 5‐point Likert scale, together with the exclusion of participants who did not use TikTok at all (i.e., participants who would have responded “never” to our self‐report question), likely compressed variability and may have constrained the detection of moderation effects (Aguinis 1995; Aguinis and Whitehead 1997). Third, the sample was predominantly comprised of women, which limits generalizability and may interact with documented gender differences in reporting accuracy, wherein females might overestimate social media use more than males (Boyle et al. 2022). Fourth, the study focused exclusively on TikTok, a platform structured around algorithmically delivered short‐form video, and correspondence between subjective and objective indicators may differ across social media platforms (Boyle et al. 2022; Irmer and Schmiedek 2023). Fifth, the sample size was modest for detecting interaction effects, particularly in moderation models, which may have limited statistical power. Sixth, self‐reported use was assessed using a Likert‐type frequency rating—a more comprehensive study would also have examined open‐ended time estimates. Seventh, most of our sample was comprised of individuals with an anorexia nervosa diagnosis, and our findings may not generalize to other eating disorders. Eighth, participants' diagnostic statuses were based on self‐reports—confirming these diagnoses via clinical interview would provide greater confidence in their validity.

## 5 Conclusion

In conclusion, this study provides evidence that Likert‐type frequency ratings of TikTok use show moderate‐to‐strong correspondence with objectively extracted behavioral indicators, suggesting that such measures can capture relative differences in general platform engagement. At the same time, substantial dispersion in objective activity highlights the limits of single‐item self‐reports as indicators of magnitude. Importantly, eating disorder diagnosis and symptom severity did not moderate the association between subjective and objective use, indicating that, within the constraints of the present design, reporting accuracy was not systematically impaired by eating disorder status. Methodologically, this study extends prior validation research by extracting and analyzing multiple forms of behavioral data directly from TikTok rather than relying on device‐level summaries such as screen time or manually processed screenshots (Ohme et al. 2020; Coyne et al. 2023). By operationalizing objective use across exposure and engagement metrics, including videos delivered, likes, sessions, and binge scrolling, the study offers a more comprehensive representation of platform activity. Together, these findings contribute to ongoing debates about the criterion validity of social media self‐report frequency measures and clarify the contexts in which these measures may be informative.

## Author Contributions

**Scott Griffiths:** conceptualization, data curation, methodology, formal analysis, funding acquisition, methodology, project administration, resources, supervision, validation, visualization, writing – original draft preparation, writing – review and editing. **Emma Halliwell:** formal analysis, visualization, writing – original draft preparation, writing – review and editing. **Harish Kannan:** data curation, methodology, software, validation, visualization, writing – review and editing. **Ben Stone:** data curation, software, validation, writing – review and editing. **Wesley Grey:** validation, writing – review and editing. **Noa Brittain:** writing – review and editing. **Eden Shaveet:** writing – review and editing. **Emily Harris:** data curation, investigation, methodology, project administration, software, validation, writing – review and editing.

## Lived Experience Involvement Statement

No specific efforts were undertaken to involve persons with lived experience in the study design or execution, or in the preparation of this manuscript.

## Funding

Scott Griffiths receives funding from the National Health and Medical Research Council and the Medical Research Future Fund (grant numbers: 1179321, 1193738).

## Ethics Statement

All procedures were approved by the Human Research Ethics Committee at the University of Melbourne: ethics ID #23072. Informed consent was obtained from all participants.

## Conflicts of Interest

Scott Griffiths has provided unpaid expertise to Government bodies in Australia, Singapore, and the United Kingdom; unpaid expertise to Meta; paid expertise to TikTok; and a combination of paid and unpaid expertise to education‐ and mental health‐related organizations in Australia. The authors affirm that no external parties or organizations had input into the design, conduct, analysis, or interpretation of this study or manuscript.

## Data Availability

Data are available at https://osf.io/btduy/overview?view_only=0d05d09d6f3e45958466132e1e793923.
